# Evaluation of Thermal Indices as the Indicators of Heat Stress in Dairy Cows in a Temperate Climate

**DOI:** 10.3390/ani11082459

**Published:** 2021-08-21

**Authors:** Geqi Yan, Hao Li, Zhengxiang Shi

**Affiliations:** 1 College of Water Resources & Civil Engineering, China Agricultural University, Beijing 100083, China; yangeqi@cau.edu.cn (G.Y.); leehcn@hotmail.com (H.L.); 2 Key Laboratory of Agricultural Engineering in Structure and Environment, Ministry of Agriculture and Rural Affairs, Beijing 100083, China; 3 Beijing Engineering Research Center on Animal Healthy Environment, Beijing 100083, China

**Keywords:** thermal index, dairy cattle, heat stress

## Abstract

**Simple Summary:**

When the ambient temperature exceeds the upper limit of a certain temperature range, heat stress is triggered and then negatively affects the production, reproduction, health, and welfare of livestock animals. Due to the limitations of ambient temperature alone as a representative measure of the thermal environment, heat stress is commonly assessed by thermal indices, which contain two or more environmental parameters representing the influence of heat exchanges between the animal and its environment. To understand and utilize the thermal indices better, we evaluated several thermal indices commonly used in the heat stress assessment of dairy cows. We found that the comprehensive climate index (CCI), which includes air temperature, relative humidity, wind speed, and solar radiation, showed a better relationship with the animal-based indicators (i.e., rectal temperature, skin temperature, and eye temperature) of heat stress. According to the results of this study, the CCI has the potential to replace the temperature–humidity index in quantifying the severity of heat stress in dairy cows.

**Abstract:**

Many thermal indices (TIs) have been developed to quantify the severity of heat stress in dairy cows. Systematic evaluation of the representative TIs is still lacking, which may cause potential misapplication. The objectives of this study were to evaluate the theoretical and actual performance of the TIs in a temperate climate. The data were collected in freestall barns at a commercial dairy farm. The heat transfer characteristics of the TIs were examined by equivalent air temperature change (ΔTeq). One-way ANOVA and correlation were used to test the relationships between the TIs and the animal-based indicators (i.e., rectal temperature (RT), respiration rate (RR), skin temperature (ST), and eye temperature (ET)). Results showed that the warming effect of the increased relative humidity and the chilling effect of the increased wind speed was the most reflected by the equivalent temperature index (ETI) and the comprehensive climate index (CCI), respectively. Only the equivalent temperature index for cows (ETIC) reflected that warming effect of solar radiation could obviously increase with increasing Ta. The THI and ETIC showed expected relationships with the RT and RR, whereas the CCI and ETIC showed expected relationships with the ST and ET. Moreover, CCI showed a higher correlation with RT (r = 0.672, *p* < 0.01), ST(r = 0.845, *p* < 0.01), and ET (r = 0.617, *p* < 0.01) than other TIs *(p* < 0.0001). ETIC showed the highest correlation with RR (r = 0.850, *p* < 0.01). These findings demonstrated that the CCI could be the most promising thermal index to assess heat stress for housed dairy cows. Future research is still needed to develop new TIs tp precisely assess the microclimates in cow buildings.

## 1. Introduction

Heat stress, defined as the sum of external forces acting on animals that induces an increase in core body temperature and evokes a physiological response, has a negative effect on the production, reproduction, health, and welfare of livestock animals [1,2]. Dairy cows, characterized by a large quantity of metabolic heat, are vulnerable to heat stress because of their compromised cooling capacity resulting from environmental conditions [3]. China is an agricultural country, where the dairy industry plays an important role in the agricultural economy. Statistics show that from 2000 to 2018, milk production nearly doubled in China. Not surprisingly, the challenges of heat stress are the greatest in southern China, which has a subtropical climate. However, recent studies reported that dairy cows in northern China, a region with a temperate climate, also underwent extended periods of heat stress [4,5]. To reduce economic losses, dairy producers need to precisely assess the environmental risks and need to initiate cooling in a timely manner for dairy cows before heat stress occurs.

So far, there is an academic consensus that heat stress is triggered when the ambient temperature reaches the upper critical temperature (UCT) of a dairy cow’s thermal neutral zone (TNZ) [3,6,7]. Nevertheless, the limitation of ambient temperature alone as a representative measure of the thermal environment is widely admitted. More often than not, the intensity of heat stress on dairy cattle is quantitatively estimated using the temperature–humidity index (THI). Since Thom first proposed the THI (originally called the discomfort index) in 1959 [8], this index has served as a de facto standard for the classification of thermal environments in animal transport and production situations and as a basis for environmental management decisions in hot seasons [9]. The THI considers the combined effect of air temperature and humidity. Because of different estimators for air humidity, there are three mainstream THI equations, which contain relative humidity, wet-bulb temperature, and dew point temperature, respectively. Previous studies have reported that different THI equations differed in their ability to detect heat stress [10,11]. Interestingly, previous researchers consistently used the equation containing relative humidity to identify the THI threshold where the physiological responses of dairy cows significantly changes [5,12,13]. Despite that, there is insufficient information on the differences among those THI equations.

In the last few decades, most efforts to develop new thermal indices (TIs) have been made along two lines: modified THI and apparent ambient temperature. The former is achieved by adding new environmental factors into the THI models or substituting old parameters, and the typical Tis that are often used are the adjusted THI (THIadj) and the black globe humidity index (BGHI) [14,15]. The TIs classified as apparent ambient temperature are achieved by converting the thermal effects of other environmental parameters into the equivalent thermal effect of air temperature, and the typical examples include the equivalent temperature index (ETI) [16], the tcomprehensive climate index (CCI) [17], and the equivalent temperature index for dairy cattle (ETIC) [18]. Previous researchers have reported the index performance under the climatic conditions they studied and have recommended some of these indices. However, systematic evaluation of the existing TIs is still lacking. Potential problems will occur if a TI is applied as an environment control strategy without a detailed examination.

A TI value has to reflect the comprehensive effect produced by the sensible and latent heat exchanges between the organism and its environment. Meanwhile, the TIs should be highly associated with the physiological responses that can indicate the thermal status of an animal. For dairy cows, effective animal-based indicators include rectal temperature, respiration rate, skin temperature, and eye temperature. Rectal temperature is a predominant indicator of core body temperature, which is a gold standard and is used in 28% of heat stress assessments [1]. Respiration rate is universally recognized as an early indicator of heat stress [19]. The skin surface is the primary site for the heat exchange process, and thus, skin temperature is highly related to thermal comfort [20]. Recent research has found that eye temperature was influenced by pain and heat stress [21,22]. Moreover, eye temperature measurements show acceptable agreement with rectal temperature measurements in dairy cows [23].

The present study aimed to examine the theoretical performance of the cow-related TIs with respect to heat transfer characteristics reflected by the parameters and to evaluate the actual relationships between the TIs and the animal-based indicators of heat stress. We restricted the current study to the temperate climate conditions in northern China. Moreover, we only evaluated the TIs mentioned earlier, which means that other TIs developed for the specified animals and environments were not included.

## 2. Materials and Methods

### 2.1. Cows, Housing, and Management

The study was conducted between July and October at a commercial dairy farm in Tianjin, China. All procedures were approved by the China Agricultural University Department of Agricultural Structure and Bioenvironmental Engineering Animal Ethics Committee (Approval ID: 20200625). This study included 826 Holstein lactating cows. Of these cows, were 161 first-lactation cows (average milk yield 27 ± 8 kg/day and average days in milk 286 ± 134 at the beginning of the study), 280 were second-lactation cows (average milk yield 30 ± 13 kg/day and average days in milk 246 ± 159), and were 384 third-lactation cows (average milk yield 29 ± 14 kg/day and average days in milk 277 ± 172). The cows were housed in free-stall barns (107.5 m × 31.0 m; double-pitched roof with a gradient of 33%), which were oriented on the east–west axis and were equipped with fans (diameter of 1.0 m; air amount of 25,430 m^3^/h; spaced every 6.0 m; 2.7 m high; activated at 18 °C air temperature). The cows were milked and fed three times per day and had free access to water.

### 2.2. Environmental Parameters and Thermal Indices of Heat Stress

Air temperature (Ta) and relative humidity (RH) were measured every 10 min using a HOBO U23-001 thermometer (Onset Computer Corp., Bourne, MA, USA; accuracy of ± 0.2 °C from −40 to 70 °C and ±2.5% from 10% to 90%). Wind speed was measured every 3 min using a TSI 9565 anemometer (TSI Inc., Shoreview, MN, USA; accuracy of ±0.015 m/s from 0 to 50 m/s). Black globe temperature (Tbg) was measured every 10 min using a black globe thermometer (JantyTech Inc., Fengtai, Beijing, China; accuracy of ±0.6 °C from 15 to 40 °C). Solar radiation (SR) was measured using a TES-1333R solar power meter (TES Electrical Electronic Corp., Taipei, Taiwan, China; accuracy of 10 W/m^2^ from 0 to 2000 W/m^2^). Wet-bulb temperature (Tw) and dew point temperature (Tdp) were obtained by inputting the Ta and RH into an online calculator (www.omnicalculator.com/physics/). (accessed 20 August 2021) These environmental parameters were used to calculate the following TIs:

Temperature—humidity Index (THI):

(1) THI1=(1.8Ta+32)-(0.55 - 0.0055RH) × (1.8Ta-26)

(2) THI2=0.72 × (Ta+Tw) + 40.6

(3) THI3=Ta + 0.36Tdp+41.5

Thermal environments were classified under the categories of into no stress (THI < 72) and heat stress (THI ≥ 72), according to the THI [24].

Black Globe Humidity Index (BGHI):

(4) BGHI=Tbg + 0.36Tdp+41.5

Thermal environments were classified into the categories of no stress (BGHI ≤ 74) and heat stress (BGHI > 74) according to the BGHI [19].

Ajusted THI (THIadj):

(5) THIadj= 4.51 + [0.8Ta + (RH/100) × (Ta-14.4) + 46.4]-1.992u+  0.0068SR

Thermal environments were classified into the categories of no stress (THIadj ≤ 74) and heat stress (THIadj > 74) according to the THIadj [15].

Equivalent Temperature Index (ETI):

(6) $$\mathrm{ETI}=27.88\;-\;0.456\mathrm{Ta}\;+\;{0.010754\mathrm{Ta}}^{2}-0.4905\mathrm{RH}\;+\;{0.00088\mathrm{RH}}^{2}+1.15u\;-\;{0.12644u}^{2}\;+0.019876\mathrm{Ta}\;\times \;\mathrm{RH}\;-\;0.046313\mathrm{Ta}\times u$$

Thermal environments were classified into the categories of no stress (ETI < 30) and heat stress (ETI ≥ 30) according to the ETI [25].

Comprehensive Climate Index (CCI):

(7) $$\mathrm{CCI}=\mathrm{Ta}+\mathrm{Eq}.\left(\mathrm{RH}\right)\;+\;\mathrm{Eq}.\left(u\right)\;+\;\mathrm{Eq}.\left(\mathrm{sr}\right)\;\mathrm{Eq}.\left(\mathrm{RH}\right)\;=\;\exp\left(0.00182\mathrm{RH}\;+\;{1.8\times 10}^{-5}\mathrm{Ta}\times \mathrm{RH}\right)\times \left({0.000054\mathrm{Ta}}^{2}+0.00192\mathrm{Ta}\;-\;0.0246\right)\times \left(\mathrm{RH}-30\right)\;\mathrm{Eq}.\left(u\right)=\frac{-6.56}{\exp\left\{\frac{1}{{\left(2.26u\;+\;0.23\right)}^{0.45}}\;\times \;\left[2.9\;+\;{1.14\;\times \;10}^{-6}u^{2.5}{-\log}_{0.3}{\left(2.26u\;+\;0.33\right)}^{-2}\right]\right\}}-{0.00566u}^{2}+3.33\;\mathrm{eq}.\left(\mathrm{sr}\right)=0.0076\mathrm{sr}\;-\;0.00002\mathrm{sr}\times \mathrm{Ta}+\;{0.00005\mathrm{Ta}}^{2}\times \sqrt{\mathrm{sr}}+0.1\mathrm{Ta}-2$$

Thermal environments were into the categories of no stress (CCI ≤ 25) and heat stress (CCI > 25) classified according to the CCI [17].

Equivalent Temperature Index for Dairy Cattle (ETIC):

(8) $$\mathrm{ETIC}=\mathrm{Ta}-0.0038\mathrm{Ta}\;\times \;\left(100-\mathrm{RH}\right)-{0.1173u}^{0.707\;}\times \;\left(39.2-\mathrm{Ta}\right)+{1.86\times 10}^{-4}\mathrm{Ta}\times \mathrm{sr}$$

Thermal environments were classified into the categories of no stress (ETIC < 18) and heat stress (ETIC ≥ 18) according to the ETIC [18].

**Figure 1:**
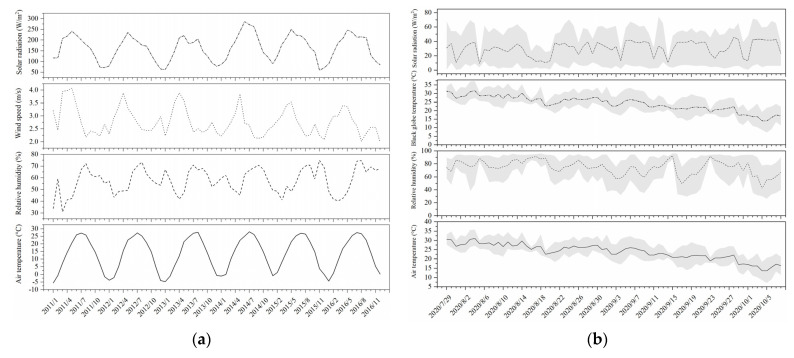
(**a**) Monthly variations in the average values of the outdoor environmental factors of the measured region (2011–2016) and (**b**) daily variations in the average values of the environmental factors inside the barns over the study period. The black line indicates the average value. The grey region indicates the maximum and minimum values.

The environmental coniditions are shown in Figure 1. The results of the environmental factors and thermal indices during this study are shown in Table 1.

### 2.3. Animal-Based Indicators of Heat Stress

**Figure 2:**
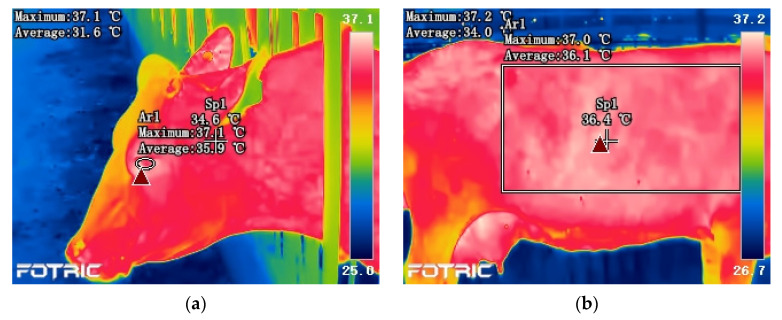
Illustration of the eye temperature (**a**) and skin temperature (**b**) measurements.

Animal measures were conducted twice daily: in the morning (0800–1200 h) and in the afternoon (1400–1800 h). Rectal temperature (RT) was measured using a veterinary digital thermometer (ShangNong Technology Inc., Qingdao, Shandong, China; accuracy of ± 0.1 °C from 35 to 39 °C). Respiration rate (RR) was measured by counting the flank movements for 60 s. Skin temperature (ST) and eye temperature (ET) were measured using a Fotric 235 infrared thermography (FOTRIC Inc., Jingan, Shanghai, China; resolution of 336 × 252 pixels; accuracy of ±2.0 °C from −20 to 150 °C). The infrared images were analyzed using AnalyzIR software (Fortic Inc., Jingan, Shanghai, China). The methods for the measurements of ST and ET using infrared thermography agreed with those from previous studies [26,27]. The Ta and RH corresponding to the time when the image was taken were inputted into the software to adjust for these variables on the camera accuracy. The emissivity was set to 0.97. Typical examples of the regions used to obtain temperature variables are shown in Figure 2. By using the shape-drawing tool, a box was placed on the trunk to obtain the ST variable, and an oval was placed on the eye to obtain the ET variable. According to the recommendations from Hoffmann et al. [23], only the maximum temperature of the region was considered in the statistical analysis. The results of the animal-based indicators during this study are shown in Table 2.

### 2.4. Statistical Analysis

#### 2.4.1. Equivalent Air Temperature Change

Under the assumption that variation in the TI value caused by a change in one environmental parameter can be offset by a change in air temperature, the equivalent air temperature change (ΔTeq) was defined as the difference between the newly generated and the original air temperature [28]. Equations (9) and (10) further explain the ΔTeq:

(9) $$\mathrm{TI}\left({\mathrm{Ta}}_{1}{,\mathrm{RH}}_{2}{,u}_{1}{,\mathrm{SR}}_{1}\right)=\mathrm{TI}\left({\mathrm{Ta}}_{2}{,\mathrm{RH}}_{1}{,u}_{1}{,\mathrm{SR}}_{1}\right)\;\mathrm{for}\;a\;\mathrm{change}\;\mathrm{from}\;{\mathrm{RH}}_{1}\;\mathrm{to}\;{\mathrm{RH}}_{2}\;\;\mathrm{TI}\left({\mathrm{Ta}}_{1}{,\mathrm{RH}}_{1}{,u}_{2}{,\mathrm{SR}}_{1}\right)=\mathrm{TI}\left({\mathrm{Ta}}_{2}{,\mathrm{RH}}_{1}{,u}_{1}{,\mathrm{SR}}_{1}\right)\;\mathrm{for}\;a\;\mathrm{change}\;\mathrm{from}\;u_{1}\;\mathrm{to}\;u2_{\;}\;\mathrm{TI}\left({\mathrm{Ta}}_{1}{,\mathrm{RH}}_{2}{,u}_{1}{,\mathrm{SR}}_{2}\right)=\mathrm{TI}\left({\mathrm{Ta}}_{2}{,\mathrm{RH}}_{1}{,u}_{1}{,\mathrm{SR}}_{1}\right)\;\mathrm{for}\;a\;\mathrm{change}\;\mathrm{from}\;{\mathrm{SR}}_{1}\;\mathrm{to}\;{\mathrm{SR}}_{2}$$

(10) $${\Delta \mathrm{Teq}=\mathrm{Ta}}_{2}{-\mathrm{Ta}}_{1}$$

A positive ΔTeq indicates a warming effect caused by the changed parameter, whereas a negative ΔTeq indicates a chilling effect. A larger absolute ΔTeq values implies a stronger warming/chilling effect in the corresponding TI.

#### 2.4.2. Analysis of Variance

One-way analysis of variance (ANOVA) was used to test the effect of different TI levels on the animal-based indicators using the following model:

(11) $$Y_{\mathrm{ij}}=\mu +{\mathrm{TI}}_{i}{\;+\;\varepsilon }_{\mathrm{ij}}$$

where Y_ij_ indicates the jth observation of the animal-based indicator; μ indicates the overall mean; ε_ij_ indicates the random error; TI_i_ indicates the effect of ith TI (both THI1 and BGHI contain sixteen levels, and i is equal to 58, 60, 62, …, 88; both THI2 and THI3 contain fifteen levels, and i is equal to 58, 60, 62,…, 86; THIadj contains fifteen levels, and i is equal to 60, 62, 64,…, 88; both ETI and CCI contain fifteen levels, and i is equal to 15, 17, 19, …, 41; ETIC contains eleven levels, and i is equal to 9, 11, 13,…, 29).

When the *p*-value from the ANOVA was less than 0.05, a post hoc test was conducted based on Fisher’s least significant difference (LSD) criterion.

#### 2.4.3. Correlation Analysis

Pearson correlation coefficients (r) between the TIs and the animal-based indicators were calculated and were then interpreted as follows: a coefficient that is less than 0.3 indicates a weak relationship; a coefficient that varies between 0.3 and 0.5 indicates a medium relationship; a coefficient that varies between 0.5 and 0.8 indicates a strong relationship; a coefficient that is more than 0.8 indicates a very strong relationship.

Comparison of the two overlapping dependent correlations (r.(TI1 and Y) vs. r.(TI2 and Y)) was tested by Hotelling’s t statistic [29]. The null hypothesis is that the correlations are the same.

Statistical analyses were performed using SPSS statistical software (IBM Corp., Armonk, NY, USA).

## 3. Results

### 3.1. Equivalent Ambient Temperature Change

#### 3.1.1. Relative Humidity

**Figure 3:**
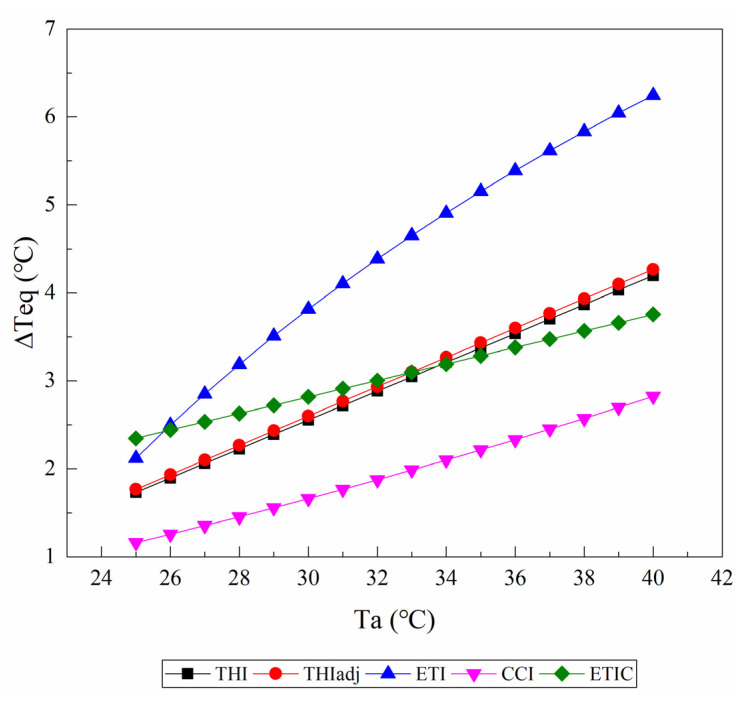
The changes of the equivalent ambient temperature change (ΔTeq) caused by an increase in relative humidity from 40% to 60% as the air temperature (Ta) rises from 25 to 40 °C are reported from the temperature–humidity index (THI), adjusted THI (THIadj), equivalent temperature index (ETI), comprehensive climate index (CCI), and equivalent temperature index for dairy cattle (ETIC). Wind speed is assumed to be 0.2 m/s, and solar radiation is assumed to be 0 W/m^2^. THI2, THI3, and black globe humidity index (BGHI) are not included since they do not contain the parameter of relative humidity.

Figure 3 shows the changes of ΔTeq caused by an increase in RH from 40% to 60% as the Ta rises from 25 to 40 °C at a wind speed of 0.2 m/s and solar radiation of 0 W/m^2^. All of the TIs treated an increase in RH as a warming effect, and the ΔTeq values increased with the Ta rising. Within a range of 25 °C to 40 °C Ta, the highest ΔTeq value was generally given by the ETI, and the smallest was given by the CCI. There were almost no differences in the value of ΔTeq and its change with the Ta between the THI1 and the THIadj. When the Ta was more than approximately 33 °C, the ETIC showed a smaller ΔTeq than the THI1.

#### 3.1.2. Wind Speed

**Figure 4:**
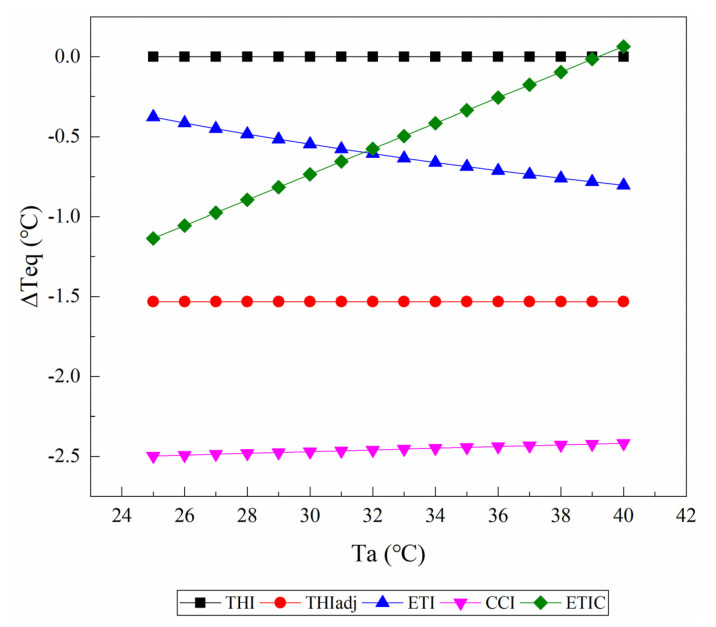
The changes of the equivalent ambient temperature change (ΔTeq) caused by an increase in wind speed from 1 m/s to 2 m/s as air temperature (Ta) rises from 25 to 40 °C are reported from the temperature–humidity index (THI), adjusted THI (THIadj), equivalent temperature index (ETI), comprehensive climate index (CCI), and equivalent temperature index for dairy cattle (ETIC). Relative humidity is assumed to be 50%, and solar radiation is assumed to be 0 W/m^2^. THI2, THI3, and black globe humidity index (BGHI) are not included since they do not contain wind speed and relative humidity.

Figure 4 shows the changes of the ΔTeq value caused by an increase in wind speed from 1 m/s to 2 m/s as the Ta rises from 25 to 40 °C at a relative humidity of 50% and solar radiation of 0 W/m^2^. With the exception of THI1, all of the TIs treated an increase in wind speed as a chilling effect when Ta was below 39 °C. For the THIadj, the ΔTeq values were constant at −1.5 °C. Similarly, the ΔTeq values given by the CCI were almost kept constant (−2.5 °C), although they decreased when the Ta increased. The ΔTeq values given by the CCI decreased as the Ta increased, but those given by the ETIC increased as the Ta increased.

#### 3.1.3. Solar Radiation

**Figure 5:**
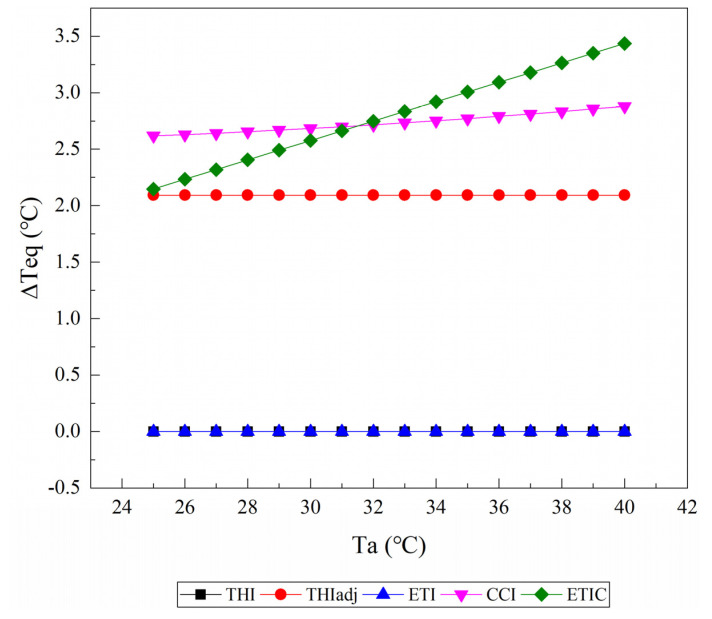
The changes of the equivalent ambient temperature change (ΔTeq) caused by an increase in solar radiation from 100 W/m^2^ to 500 W/m^2^ as air temperature (Ta) rises from 25 to 40 °C are reported from the temperature–humidity index (THI), adjusted THI (THIadj), equivalent temperature index (ETI), comprehensive climate index (CCI), and equivalent temperature index for dairy cattle (ETIC). Relative humidity is assumed to be 50%, and wind speed is assumed to be 0.2 m/s. THI2, THI3, and black globe humidity index (BGHI) are not included since they do not contain solar radiation and relative humidity.

Figure 5 shows the changes of the ΔTeq values caused by an increase in solar radiation from 100 W/m^2^ to 500 W/m^2^ at a wind speed of 0.2 m/s and a relative humidity of 50%. THI1 and ETI did not include the parameter of solar radiation, and thus, their ΔTeq values were equal to zero. Other TIs (i.e., THIadj, CCI, and ETIC) treated an increase in solar sr as a warming effect. For the THIadj, the ΔTeq values were constant at approximately 2.1 °C. The ΔTeq values obtained from the ETIC decreased with when the Ta increased, while those obtained from the CCI decreased as the Ta increased at a comparatively slower rate.

### 3.2. Effect of the TI Values on the Animal-Based Indicators

#### 3.2.1. Rectal Temperature

**Figure 6:**
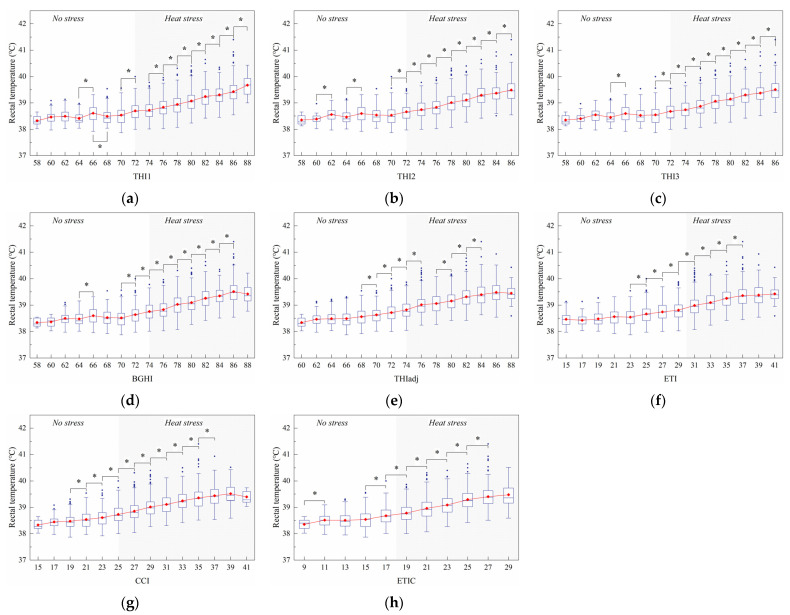
Effect of the thermal index values on rectal temperature. The interquartile range (IQR) is shown by a blue box, the median as a blue horizontal line, the mean as a red square, and the whiskers extend to 1.5 IQR. Outliers are indicated by blue dots. An asterisk indicates a significant (*p* < 0.05) change in the variable. The thermal indices are the temperature–humidity index (THI1 (**a**), THI2 (**b**), THI3 (**c**)), black globe humidity index (BGHI (**d**)), adjusted THI (THIadj (**e**)), equivalent temperature index (ETI (**f**)), comprehensive climate index (CCI (**g**)), and equivalent temperature index for dairy cattle (ETIC (**h**)).

Figure 6 shows the changes in ther rectal temperature with the index value. Figure 6a shows that nine significant (*p* < 0.05) increases in RT were observed at 64, 70, 74, 76, 78, 80, 82, 84, and 86 THI1. There was a significant (*p* < 0.05) decrease in RT at 66 THI1. Figure 6b shows that ten significant (*p* < 0.05) increases in RT were observed at 60, 64, 70, 72, 74, 76, 78, 80, 82, and 84 THI2. The insignificant (*p* = 0.076) difference in RT was found between 60 and 62 THI3, although the changes of the RT with THI3 were similar to those with THI2 (Figure 6c). Figure 6d shows that nine significant (*p* < 0.05) increases in RT were found at 64, 70, 72, 74, 76, 78, 80, 82, and 84 BGHI. Likewise, nine significant (*p* < 0.05) increases in RT were reported at 19, 21, 23, 25, 27, 29, 31, 33, and 35 CCI (Figure 6g). However, only seven significant (*p* < 0.05) increases in RT were found at 68, 70, 72, 74, 78, 80, and 82 THIadj (Figure 6e), 23, 25, 27, 29, 31, 33, and 35 ETI (Figure 6f), and 9, 15, 17, 19, 21, 23, and 25 ETIC (Figure 6h), respectively.

#### 3.2.2. Respiration Rate

**Figure 7:**
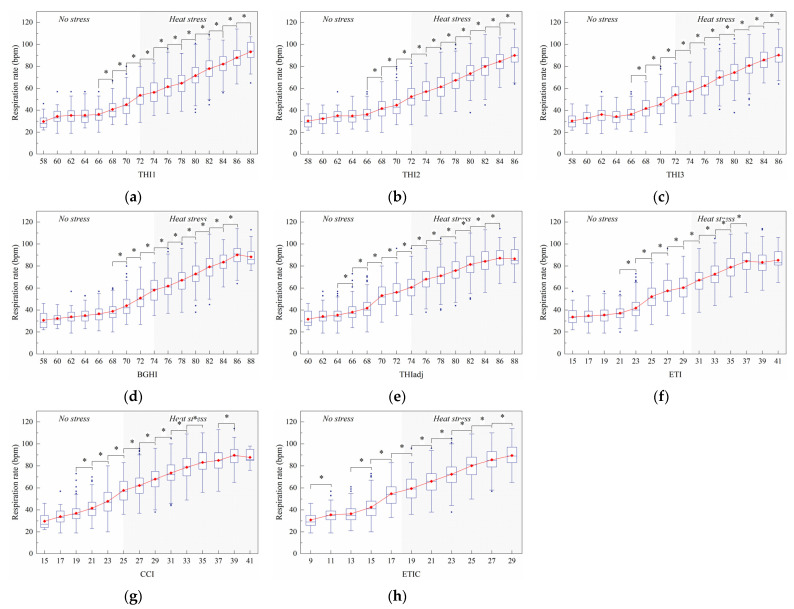
Effect of the thermal index values on respiration rate. The interquartile range (IQR) is shown by a blue box, the median as a blue horizontal line, the mean as a red square, and the whiskers extend to 1.5 IQR. Outliers are indicated by blue dots. An asterisk indicates a significant (*p* < 0.05) change in the variable. The thermal indices are temperature–humidity index (THI1 (**a**), THI2 (**b**), THI3 (**c**)), black globe humidity index (BGHI (**d**)), adjusted THI (THIadj (**e**)), equivalent temperature index (ETI (**f**)), comprehensive climate index (CCI (**g**)), and equivalent temperature index for dairy cattle (ETIC (**h**)).

Figure 7 shows the changes of the respiration rate with the index value. A total of eleven significant (*p* < 0.05) and continuous increases in RR were found from 66 to 86 THI1 (Figure 7a) and from 64 to 84 THIadj (Figure 7e). There were ten significant (*p* < 0.05) and continuous increases in RR that were found from 66 to 84 for both THI2 (Figure 7b) and THI3 (Figure 7c). Similarly, nine significant (*p* < 0.05) and continuous increases in RR were observed from 68 to 84 BGHI (Figure 7d). From Figure 7f, eight significant (*p* < 0.05) and consecutive increases in RR were observed from 21 to 35 ETI. Figure 7g shows that nine significant (*p* < 0.05) increases in RR were observed at 19, 21, 23, 25, 27, 29, 31, 33, and 37 CCI. With the exception of at 11 ETIC, nine significant (*p* < 0.05) increases in RR were found from 9 to 27 ETIC.

#### 3.2.3. Skin Temperature

**Figure 8:**
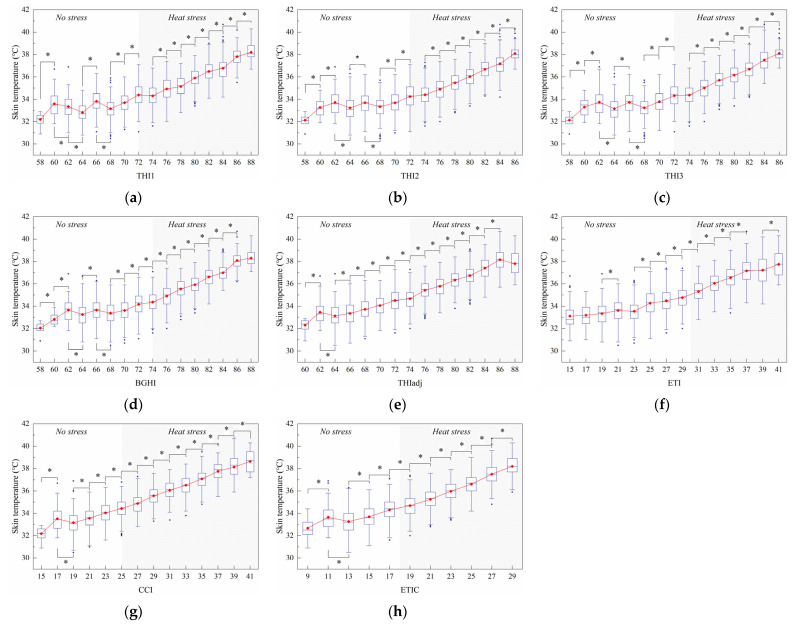
Effect of the thermal index values on skin temperature. The interquartile range (IQR) is shown by a blue box, the median as a blue horizontal line, the mean as a red square, and the whiskers extend to 1.5 IQR. Outliers are indicated by blue dots. An asterisk indicates a significant (*p* < 0.05) change in the variable. The thermal indices are the temperature–humidity index (THI1 (**a**), THI2 (**b**), THI3 (**c**)), black globe humidity index (BGHI (**d**)), adjusted THI (THIadj (**e**)), equivalent temperature index (ETI (**f**)), comprehensive climate index (CCI (**g**)), and equivalent temperature index for dairy cattle (ETIC (**h**)).

Figure 8 shows the change of the skin temperature with the index value. Figure 8a shows that eleven significant (*p* < 0.05) increases in ST were observed at 58, 64, 68, 70, 74, 76, 78, 80, 82, 84, and 86 THI1, and three significant (*p* < 0.05) decreases in ST were observed at 60, 62, and 66 THI1. For THI2, THI3, and BGHI, ST significantly (*p* <0.05) increased at the 58, 60, 64, 68, 70, 74, 76, 78, 80, 82, 84, and 86 THI values and decreased at the 62 and 66 THI values (Figure 8b–d). According to Figure 8e, ST at 62 THIadj were significantly (*p* < 0.05) higher than that at 60 and 64 THIadj. There were eleven significant (*p* < 0.05) and continuous increases in ST that were observed from 64 to 84 THIadj. Figure 8f shows that nine significant (*p* < 0.05) increases in ST were reported at 19, 23, 25, 27, 29, 31, 33, 35, and 39 ETI. Apart from one significant decrease observed at 17 CCI, twelve significant (*p* < 0.05) increases in ST were found from 15 to 39 CCI (Figure 8g). Likewise, there were nine significant (*p* < 0.05) increases in ST observed from 9 to 27 ETIC, apart from one decrease observed at 11 ETIC (Figure 8h).

#### 3.2.4. Eye Temperature

**Figure 9:**
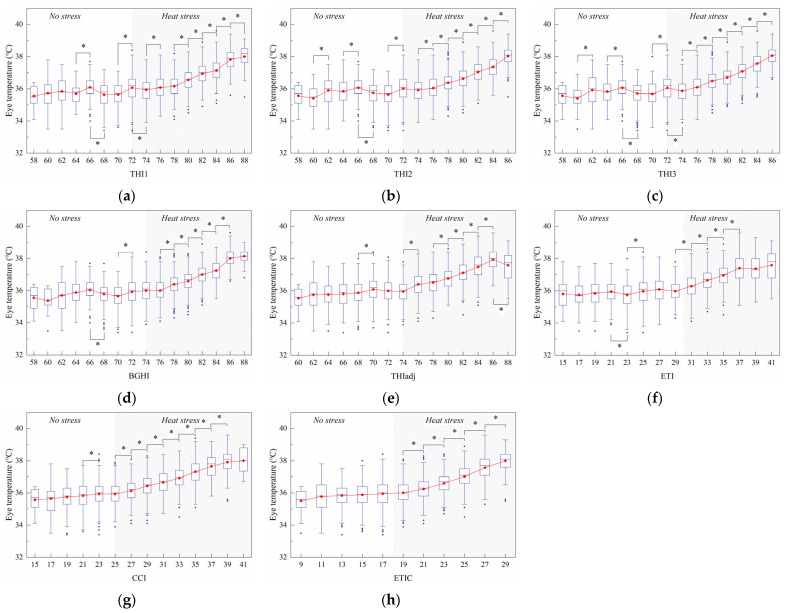
Effect of the thermal index values on eye temperature. The interquartile range (IQR) is shown by a blue box, the median as a blue horizontal line, the mean as a red square, and the whiskers extend to 1.5 IQR. Outliers are indicated by blue dots. An asterisk indicates a significant (*p* < 0.05) change in the variable. The thermal indices are the temperature–humidity index (THI1 (**a**), THI2 (**b**), THI3 (**c**)), black globe humidity index (BGHI (**d**)), adjusted THI (THIadj (**e**)), equivalent temperature index (ETI (**f**)), comprehensive climate index (CCI (**g**)), and equivalent temperature index for dairy cattle (ETIC (**h**)).

Figure 9 shows the changes of the eye temperature with the index values. Figure 9a shows that eight significant (*p* < 0.05) increases in ET were found at 64, 70, 74, 78, 80, 82, 84, and 86 THI1, and two significant (*p* < 0.05) decreases in ET were found at 66 and 72 THI1. Figure 9b shows that nine significant (*p* < 0.05) increases in ET were observed at 60, 64, 70, 74, 76, 78, 80, 82, and 84 THI2, and one significant (*p* < 0.05) decrease in ET was observed at 66 THI2. Compared to the results from THI2, one extra significant (*p* < 0.05) decrease in ET was observed at 72 THI3 (Figure 9c). A significant (*p* < 0.05) decrease in ET occurred at 66 BGHI, and then six significant (*p* < 0.05) increases in ET occured at 70, 76, 78, 80, 82, and 84 BGHI (Figure 9d). There were six significant (*p* < 0.05) decreases in ET observed at 68, 74, 78, 80, 82, and 84 THIIadj, but one significant (*p* < 0.05) decrease in ET occurred at 86 THIadj (Figure 9e). Figure 9f shows that one significant (*p* < 0.05) decrease in ET occurred at 21 ETI, and then five significant (*p* < 0.05) increases in ET occurred at 23, 29, 31, 33, and 35 ETI. Figure 9g shows that the first significant (*p* < 0.05) increase in ET was found at 21 CCI and then seven significant (*p* < 0.05) and consecutive increases in ET were found from 25 to 37 CCI. According to Figure 9h, five significant (*p* < 0.05) and continuous increases in ET were observed from 19 to 27 ETIC.

### 3.3. Correlations between Indices and Animal-Based Indicators

Table 3 presents that the correlations among the TIs were positive and very strong (r ≥0.95), and the correlations between the TIs and the animal-based indicators were positive and high (r ≥ 0.5). Results of the correlation comparison are listed in Table 4. The CCI showed the highest correlation with rectal temperature (r = 0.672, *p* < 0.01), followed by the THIadj (r = 0.667, *p* < 0.01; r.CCI > r.THIadj, *p* < 0.0001) and the ETIC (r = 0.662, *p* < 0.01; r.CCI > r.ETIC, *p* < 0.0001). The ETIC showed the highest correlation with respiration rate (r = 0.850, *p* < 0.01), followed by the THI3 (r = 0.847, *p* < 0.01; r.ETIC > r.THI3, *p* = 0.0793) and the BGHI (r = 0.846, *p* < 0.01; r.ETIC > r.BGHI, *p* = 0.0274). The CCI exhibited the highest correlation with skin temperature (r = 0.845, *p* < 0.01), followed by the THIadj (r = 0.827, *p* < 0.01; r.CCI > r.THIadj, *p* < 0.0001) and the ETIC (r = 0.820, *p* < 0.01; r.CCI > r.ETIC, *p* < 0.001). Additionally, the CCI presented the highest correlation with eye temperature (r = 0.617, *p* < 0.01), followed by the BGHI (r = 0.598, *p* < 0.01; r.CCI > r.BGHI, *p* = 0.0001) and the ETIC (r = 0.592, *p* < 0.01; r.CCI > r.ETIC, *p* < 0.0001).

## 4. Discussion

The aim of the present study was to evaluate the performance of the TIs with respect to their heat transfer characteristics and relationships with the animal-based indicators. We calculated the equivalent ambient temperature change that resulted from the changed relative humidity, wind speed, and solar radiation. The results of this study indicate that the warming effect of the increased RH was the most reflected by the ETI, and the chilling effect of the increased wind speed was the most reflected by the CCI. These results could be explained by the environmental conditions that were used to develop the model. According to Baeta et al. [16], the ETI was developed based on variable environments in a climatic chamber with Ta ranging from 16 °C to 41 °C, RH ranging from 40% to 90%, and wind speed ranging from 0.5 m/s to 6.5m/s. However, the authors did not specifically emphasize the significance of RH. Compared to the relationship between the ETI and RH, the relationship between the CCI and wind speed seems clearer. According to Mader et al. [17], the wind chill index (WCI), which defines the relationship between wind speed and Ta, was used as the basis for modeling the CCI under cold environments (Ta < 5 °C). Although the warming effect of increased solar radiation was indicated by the THIadj, CCI, and ETIC, only the ETIC reflected that the warming effect of solar radiation could apparently increase with increasing Ta. This finding can be demonstrated by Wang et al. [18] since the authors specifically highlighted the interaction between Ta and other environmental parameters (i.e., RH, wind speed, and solar radiation) in the process of modeling the ETIC. For more complex heat transfer characteristics, Bjerg et al. [30] investigated the changes of the chilling effect of wind speed with increasing wind speed. In addition, Wang et al. [28] explored the changes in the chilling effect of wind speed with increasing RH. In this study, we only focused on the interaction between the warming or chilling effect caused by one changed environmental variable and the air temperature. It has been confirmed that evaporation is the most important mechanism that is automatically exhibited by cattle to strengthen heat loss in hot environments [31]. Evaporative heat loss consists of respiratory and cutaneous heat loss, and the latter was governed by the moisture gradient between the ambient air and the skin surface [31]. Under high Ta and RH conditions, restricted cutaneous evaporation exacerbates heat stress, which can be recognized by the TIs containing the RH parameter (i.e., THI, THIadj, ETI, CCI, and ETIC). Under high Ta and wind speed, the sweating rate is a greater driving force for cutaneous evaporation than wind speed, which means that the chilling effect of wind speed decreases with the increasing Ta. This fact was recognized by the CCI but was significantly reflected by the ETIC. It should be noted that a higher relative significance to a certain parameter or a better representation of the interaction between Ta and the other parameters is not fully equivalent to a better TI performance in actual conditions.

Further, we examined the performance of the TIs through their relationship with the physiological responses of dairy cows. As mentioned above, all of the animal-based indicators (i.e., RT, RR, ST, and ET) used in this study have been proven to be useful and effective for heat stress assessment in dairy cows by previous literature. Generally, the values of the animal-based indicators increased with the increases in heat stress magnitude. For RT, it was expected that the RT underwent a steady phase and then rose as the index value rose [5,13]. However, there were some fluctuations in RT under the no-stress conditions indicated by THI1. The expected relationship that RT remains stable under no-stress conditions and rises significantly, continuously, and linearly under heat stress conditions was better reflected by THI2, THI3, and ETIC. We noticed that the RT within the extreme heat stress level was lower (no significantly) than that within the prior heat stress level. This unexpected result was associated with the small sample size within the highest level group. The changes of the RR with the index value were generally consistent with the expectation that the RR rose slowly and then rapidly with the increase in the heat stress level. All of the TIs evaluated in this study reflected that the RR grew significantly and continuously with the increase in the index value. However, the relationship was better reflected by the THI1, THI2, and ETIC. Interestingly, the first significant increase in RR was found at 66 THI1 and THI2, which is in accordance with the critical THI threshold identified for RR from the recent studies [13]. With respect to ST, an ideal relationship could be that the ST grew approximately linearly with the increasing index value, as is to be expected for RR. Based on this, the relationships of the ST with the CCI and ETIC corresponded to the expectation. One unanticipated result was that the ST significantly decreased at 17 CCI and 11 ETIC. This can likely be attributed to the fact that the ST was measured using infrared thermography and that dirt, moisture, or other secondary factors (e.g., contact with the ground while lying) can alter the emissivity and conductivity and thus can cause inaccurately measured results [32]. Compared to ST, ET is less likely to be affected by the factors mentioned above and commonly serves as a proxy for internal temperature [26]. In the current evaluation, the ET fluctuated with the values of the THI1, THI2, THI3, BGHI, and ETI under the no-stress conditions. Additionally, the relationship between ET and THIadj was unsatisfactory because of the inconsecutive increase and unexpected decrease in ET under the heat-stress conditions indicated by the THIadj. Obviously, in comparison to other TIs, the CCI and ETIC performed better with respect to their relationship with ET.

Further, correlation, which is one of the methodologies commonly used in studies for the evaluation of thermal indices, was conducted to examine the relationships between the TIs and animal-based indicators. In the current study, we investigated and compared the correlations among the indices. We found that the RT, ST, and ET correlated the most closely with the CCI. High correlations of CCT with ST and ET agreed with another study conducted in southern China (Yancheng, Jiangsu, China), which reported that the CCI showed higher correlations (0.443 ≤ r ≤ 0.849) with the body surface temperature than other TIs (e.g., THI1, BGHI, THIadj, ETI, ETIC) [33]. Consistent with the present study, a study from Da Silva et al. [34], in which the data were collected from a tropical environment, reported that the CCI showed a higher correlation (r = 0.374) with RT than the BGHI and other TIs (e.g., the heat load index (HLI) and the index of thermal stress for cows (ITSC)). We also observed the highest correlation with RR shown by the ETIC. This result may be explained by the fact that RR was used for the response variable to develop the ETIC regression model. Additionally, the original authors stated that the ETIC (r = 0.703) performed better than the CCI (r = 0.692) and THIadj (r = 0.671) in terms of RR [18]. With the use of different data sets the correlation results can vary. For example, a previous study also reported that ETI was significantly correlated with the RT (r = 0.293) and RR (r = 0.520) of dairy cattle in the pasture and recommended it as the best index for heat stress evaluation in tropical environments [25]. Li et al. [35] evaluated eight Tis using two data sets; they reported that the ETI showed the highest correlation with RR (r = 0.34) based on the first data set collected in three breeds of dairy cow and six breeds of feedlot heifer from five regions in the United States, and the BGHI correlated the most closely with the RR (r = 0.73) and ST (r = 0.56) based on the second data set generated from a four-day measurement in twelve Holsteins in climate-controlled chambers. Despite the good performance of some data sets, there were some doubts over the ETI since it was developed on the basis of limited animals and short treatment observation periods (3 days) [36].

To sum up the results in this study, we found that the CCI and ETIC were the two best TIs for heat stress assessment. CCI performed better with respect to its relationships with the physiological responses (i.e., the changes and correlations of RT, ST, and ET with the CCI). For precision livestock farming, the main drawback of the CCI concerning the complexity of computation can be overcome when the algorithm is inserted into the environment forewarning and controlling systems in animal housings. ETIC mainly performed well in aspects of heat transfer characteristics and correlations with RR. Moreover, ETIC includes four main factors driving heat stress, and it can be calculated relatively conveniently. Combining the findings of our previous study in southern China, we found that CCI has a satisfactory performance in assessing the heat stress of dairy cows kept in semi-confined housing systems in China. Cows are housed in free-stall barns where there is no ambient temperature regulation. The barns are typically uninsulated and are naturally ventilated with curtain sidewalls. Cooling systems such as sprinklers and panel fans are included and are only used in hot seasons to alleviate heat stress. Solar radiation has an indirect effect on animal heat stress by heating the enclosure structure of the housings. Indoor airflow is usually turbulent and is governed by outside wind, the difference between inside and outside Ta, and fans.

Caution must be applied when the findings of this study are extrapolated to other situations. The final results of index evaluation can also be influenced by other factors, including breeds (i.e., Bos taurus, Bos indicus, and water buffalo), regional climates (i.e., tropical, subtropical, and temperate climates), production systems (i.e., free-range and confined housing), and heat stress acclimation [37]. Prior studies have reported that Ta could provide similar performance in assessing heat stress compared to other TIs [11,38]. Nevertheless, there is no doubt that Ta can not represent the overall environmental stress forced upon dairy cows. Objectively, these studies reveal that some issues still exist in the current TIs. As far as we know, to date, the existing TIs are all the same type of model—an empirical model. Previous experts seemingly paid too much attention to seeking the statistical association between animal-based indicators and multi environmental factors. Future work regarding developing novel TIs should be oriented towards the essence of thermal stress—an imbalance between heat production and loss.

## 5. Conclusions

This study evaluated the thermal indices applied for heat stress assessment in dairy cows in a temperate climate in northern China. Compared to other investigated indices, the comprehensive climate index (CCI) performed better due to its relationships with the rectal temperature, skin temperature, and eye temperature. The equivalent temperature index for dairy cattle (ETIC) mainly performed better in regard to the heat transfer characteristics and the correlation with the respiration rate. The evaluation of the results of the thermal indices could be influenced by animal and environmental factors. Nevertheless, the current study demonstrated the findings from previous reports that the CCI could be the most promising thermal index to assess heat stress for housed dairy cows. Moreover, there is still a real need to develop new thermal indices for precision environment control of livestock buildings.

## Figures and Tables

**Table 1:** Descriptive statistics for the environmental factors and thermal indices during this study.

Item	Number	Minimum	Maximum	Mean	Standard Deviation
Air temperature (°C)	3005	13.55	36.00	26.33	4.72
Relative humidity (%)	3005	27.57	91.70	69.75	15.89
Black globe temperature (°C)	3005	13.55	36.70	26.93	4.86
Wind speed (m/s)	3005	0	4.50	2.50	0.90
Solar radiation (W/m^2^)	3005	0	64.30	24.68	13.42
Temperature–humidity index (THI1) ^1^	3005	56.76	87.48	75.68	6.87
Temperature–humidity index (THI2) ^1^	3005	57.08	86.31	75.35	6.35
Temperature–humidity index (THI3) ^1^	3005	57.02	86.44	74.99	6.28
Black globe humidity index (BGHI)	3005	57.02	87.70	75.59	6.38
Ajusted THI (THIadj)	3005	59.30	89.23	75.34	6.47
Equivalent tempeprature index (ETI)	3005	14.18	41.64	28.93	6.12
Comprehensive climate index (CCI)	3005	13.91	40.13	27.16	5.62
Equivalent temperature index for dairy cattle (ETIC)	3005	8.40	29.68	20.63	4.76

^1^ THI1, THI2, and THI3 contain relative humidity, wet-bulb temperature, and dew point temperature, respectively.

**Table 2:** Descriptive statistics for the animal-based indicators during this study.

Item	Number	Minimum	Maximum	Mean	Standard Deviation
Rectal temperature (°C)	3005	37.87	41.4	38.92	0.47
Respiration rate (bpm)	3005	19	114	62.77	19.34
Skin temperature (°C)	2996	30.5	40.7	35.23	1.67
Eye temperature (°C)	2924	33.4	39.6	36.41	0.96

**Table 3:** Pearson correlation coefficients between thermal indices and animal-based indictors.

Variable	Statistic	THI1	THI2	THI3	BGHI	THIadj	ETI	CCI	ETIC	RT	RR	ST	ET
**THI1**	r	1	0.998	0.996	0.995	0.966	0.987	0.947	0.984	0.643	0.843	0.793	0.572
	*p*		<0.01	<0.01	<0.01	<0.01	<0.01	<0.01	<0.01	<0.01	<0.01	<0.01	<0.01
**THI2**	r		1	0.999	0.996	0.962	0.980	0.943	0.985	0.640	0.844	0.792	0.574
	*p*			<0.01	<0.01	<0.01	<0.01	<0.01	<0.01	<0.01	<0.01	<0.01	<0.01
**THI3**	r			1	0.999	0.961	0.974	0.946	0.984	0.645	0.847	0.801	0.586
	*p*				<0.01	<0.01	<0.01	<0.01	<0.01	<0.01	<0.01	<0.01	<0.01
**BGHI**	r				1	0.961	0.970	0.948	0.982	0.649	0.846	0.809	0.598
	*p*					<0.01	<0.01	<0.01	<0.01	<0.01	<0.01	<0.01	<0.01
**THIadj**	r					1	0.980	0.994	0.990	0.667	0.837	0.827	0.586
	*p*						<0.01	<0.01	<0.01	<0.01	<0.01	<0.01	<0.01
**ETI**	r						1	0.958	0.984	0.640	0.828	0.782	0.546
	*p*							<0.01	<0.01	<0.01	<0.01	<0.01	<0.01
**CCI**	r							1	0.980	0.676	0.833	0.845	0.617
	*p*								<0.01	<0.01	<0.01	<0.01	<0.01
**ETIC**	r								1	0.662	0.850	0.820	0.592
	*p*									<0.01	<0.01	<0.01	<0.01
**RT**	r									1	0.741	0.681	0.574
	*p*										<0.01	<0.01	<0.01
**RR**	r										1	0.775	0.598
	*p*											<0.01	<0.01
**ST**	r											1	0.715
	*p*												<0.01
**ET**	r												1

**Table 4:** Comparison of correlations with rectal temperature (RT), respiration rate (RR), skin temperature (ST), and eye temperature (ET).

Variable	RT		RR		ST		ET	
	Hotelling’s t (df = 3002)	*p*	Hotelling’s t (df = 3002)	*p*	Hotelling’s t (df = 2993)	*p*	Hotelling’s t (df = 2921)	*p*
r.THI1 vs. r.THI2	3.3956	0.0007	−1.6156	0.1063	1.4200	0.1557	−2.0880	0.0369
r.THI1 vs. r.THI3	−1.6033	0.109	−4.6097	<0.0001	−8.2067	<0.0001	−10.5800	<0.0001
r.THI1 vs. r.BGHI	−4.3239	<0.0001	−3.0837	0.0021	−15.2100	<0.0001	−18.3000	<0.0001
r.THI1 vs. r.THIadj	−6.6583	<0.0001	2.3754	0.0176	−12.6980	<0.0001	−3.5820	0.0003
r.THI1 vs. r.ETI	1.3323	0.1829	9.4858	<0.0001	6.1263	<0.0001	10.7310	<0.0001
r.THI1 vs. r.CCI	−7.5368	<0.0001	3.1935	0.0014	−16.3540	<0.0001	−9.504	<0.0001
r.THI1 vs. r.ETIC	−7.7799	<0.0001	−4.0801	<0.0001	−14.5620	<0.0001	−7.5180	<0.0001
r.THI2 vs. r.THI3	−8.0820	<0.0001	−6.9426	<0.0001	−19.3190	<0.0001	−18.8600	<0.0001
r.THI2 vs. r.BGHI	−7.2790	<0.0001	−2.2988	0.0216	−18.3300	<0.0001	−18.9800	<0.0001
r.THI2 vs. r.THIadj	−7.2026	<0.0001	2.6328	0.0085	−12.1970	<0.0001	−2.9060	0.0037
r.THI2 vs. r.ETI	0.0000	1	8.1727	<0.0001	4.4856	<0.0001	9.2882	<0.0001
r.THI2 vs. r.CCI	−7.9281	<0.0001	3.4025	0.0007	−16.0650	<0.0001	−8.7500	<0.0001
r.THI2 vs. r.ETIC	−9.3260	<0.0001	−3.6130	0.0003	−15.6510	<0.0001	−6.9840	<0.0001
r.THI3 vs. r.BGHI	−6.4730	<0.0001	2.3047	0.0212	−17.3100	<0.0001	−19.0900	<0.0001
r.THI3 vs. r.THIadj	−5.7939	<0.0001	3.7366	0.0002	−9.0665	<0.0001	0.0000	1
r.THI3 vs. r.ETI	1.5757	0.1152	8.5904	<0.0001	7.6148	<0.0001	11.8080	<0.0001
r.THI3 vs. r.CCI	−7.0155	<0.0001	4.4672	<0.0001	−13.6980	<0.0001	−6.4790	<0.0001
r.THI3 vs. r.ETIC	−6.9551	<0.0001	−1.7555	0.0793	−10.1690	<0.0001	−2.2500	0.0245
r.BGHI vs. r.THIadj	−4.7432	<0.0001	3.3562	0.0008	−6.2982	<0.0001	2.9011	0.0037
r.BGHI vs. r.ETI	2.6504	0.0081	7.5636	<0.0001	10.2609	<0.0001	14.5390	<0.0001
r.BGHI vs. r.CCI	−6.2288	<0.0001	4.2133	<0.0001	−11.4330	<0.0001	−4.0520	0.0001
r.BGHI vs. r.ETIC	−5.0088	<0.0001	−2.2070	0.0274	−5.5448	<0.0001	2.1334	0.033
r.THIadj vs. r.ETI	9.9701	<0.0001	4.5172	<0.0001	22.6397	<0.0001	13.5500	<0.0001
r.THIadj vs. r.CCI	−6.1202	<0.0001	3.6567	0.0003	−17.2370	<0.0001	−20.4900	<0.0001
r.THIadj vs. r.ETIC	2.6003	0.0094	−9.5786	<0.0001	4.8173	<0.0001	−2.84500	0.0045
r.ETI vs. r.CCI	−9.2415	<0.0001	−1.7398	0.082	−22.6040	<0.0001	−17.1700	<0.0001
r.ETI vs. r.ETIC	−9.0234	<0.0001	−12.8430	<0.0001	−20.9370	<0.0001	−17.8300	<0.0001
r.CCI vs. r.ETIC	5.2047	<0.0001	−8.8408	<0.0001	12.8251	<0.0001	8.6128	<0.0001

## Data Availability

The data presented in this study are available from the corresponding author upon reasonable request.
